# Estimating Temperature-Mortality Exposure-Response Relationships and Optimum Ambient Temperature at the Multi-City Level of China

**DOI:** 10.3390/ijerph13030279

**Published:** 2016-03-03

**Authors:** Qiang Zeng, Guoxing Li, Yushan Cui, Guohong Jiang, Xiaochuan Pan

**Affiliations:** 1 Tianjin Centers for Disease Control and Prevention, Huayue Road, Hedong District, Tianjin 300011, China; zengqianghaiyan@126.com (Q.Z.); yushan.273@163.com (Y.C.); jiangguohongtjcdc@126.com (G.J.); 2 Department of Occupational and Environmental Health, School of Public Health, Peking University, Xueyuan Road, Haidian District, Beijing 100191, China

**Keywords:** temperature, mortality, generalized additive model, distributed lag non-linear model, lagged effects

## Abstract

Few studies have explored temperature–mortality relationships in China, especially at the multi-large city level. This study was based on the data of seven typical, large Chinese cities to examine temperature-mortality relationships and optimum temperature of China. A generalized additive model (GAM) was applied to analyze the acute-effect of temperature on non-accidental mortality, and meta-analysis was used to merge data. Furthermore, the lagged effects of temperature up to 40 days on mortality and optimum temperature were analyzed using the distributed lag non-linear model (DLNM). We found that for all non-accidental mortality, high temperature could significantly increase the excess risk (ER) of death by 0.33% (95% confidence interval: 0.11%, 0.56%) with the temperature increase of 1 °C. Similar but non-significant ER of death was observed when temperature decreased. The lagged effect of temperature showed that the relative risk of non-accidental mortality was lowest at 21 °C. Our research suggests that high temperatures are more likely to cause an acute increase in mortality. There was a lagged effect of temperature on mortality, with an optimum temperature of 21 °C. Our results could provide a theoretical basis for climate-related public health policy.

## 1. Introduction

In recent years, against the background of global climate change, one of the most important research topics in the field of public health is the relationship between ambient temperature and health [1]. Several lines of evidence have shown the health effects related to extreme temperatures (especially the impact of a heat wave) [2,3,4,5,6,7]. In addition, epidemiological research has been carried out in non-extreme weather conditions. Because mortality-monitoring data are well documented, researchers have preferred to explore the relationship between temperature and mortality, including both the acute impact and the lagged effect of temperature on mortality [8]. Previous studies have identified a “U-“ or “V-“ curve relationship between the temperature and the disease mortality in different regions. When the temperature is below or above a certain critical threshold, mortality gradually increases as the temperature is decreased or increased [9,10,11,12]. This demarcation point of the "U" curve is possibly the optimum temperature for the population.

Several studies have explored the exposure–response relationship between temperature and mortality in different individual cities [13,14,15]. Nonetheless, it is difficult to compare the results between single-city studies because there are differences in research design, model selection and parameter settings. The poor homogeneity between single-city studies underscores the need for multi-city studies. However, these studies have been carried out mainly in developed European and American countries. China is the world’s biggest developing country and climate change in China has gained much attention. However, few multi-city studies have focused on the relationship between temperature and mortality in China. Previous studies has been conducted on the mortality effects of heat waves, high temperature and low temperature [7,16], and the acute mortality effects of diurnal temperature range [17]. Moreover, a multi-community study was recently completed [18]. Although these studies have revealed that there is an exposure–response relationship between temperature and mortality in China, the data are flawed because they were either obtained during different time periods or were not adjusted for confounding factors such as air pollution.

Thus, it is necessary to perform a systematic study in China that explores the exposure–response relationship of temperature and mortality at a multi-city level. We selected seven cities in China (Beijing, Tianjin, Xi'an, Harbin, Shanghai, Guangzhou and Wuhan) and gathered daily meteorological monitoring data, daily air quality data and disease mortality data between 1 January 2007 and 31 December 2009. A generalized additive model (GAM) was applied to establish an expose–response curve between daily mean temperature and non-accidental mortality in China. We explored the risk of death under different temperatures and further investigated the optimum temperature for the population by combination with a distributed lag non-linear model (DLNM).

## 2. Materials and Methods

### 2.1. Data Sources

Daily meteorological monitoring data, death surveillance data and air quality monitoring data were gathered in seven Chinese cities (Beijing, Tianjin, Xi’an, Harbin, Shanghai, Guangzhou and Wuhan) between 1 January 2007 and 31 December 2009.

Meteorological monitoring data were obtained from the Meteorological Data Sharing Center, National Weather Service. Monitoring indicators included daily mean air pressure, daily mean temperature, daily mean relative humidity, and daily mean wind speed.

Air quality monitoring data were obtained from the Environmental Monitoring Center of each city. The pollutants monitored include inhalable particles (PM_10_), nitrogen dioxide (NO_2_) and sulfur dioxide (SO_2_).

Mortality surveillance data were obtained from the cause of death registration report information system of the Chinese Center for Disease Control and Prevention. The deaths of each city were sorted according to the date of death and primary cause of death. Primary cause of death was classified according to the International Classification of Diseases 10 (ICD-10). ICD-10 coding of non-accidental death were from A00 to R99.

Population data sources were obtained from the 2009 Statistical Yearbook of each city and a population of more than 55,000,000 was studied in this research. The detailed information of coverage areas and population of each city are shown in Table 1. There was 1 monitoring site distributed in each district of every study city; thus, there were 6–14 air pollution monitoring stations in each city. There was one monitoring station for meteorological data in each city.

### 2.2. Statistical Analysis

The generalized additive model (GAM) was an expansion of the traditional generalized linear model. The GAM could use different functions to fit a nonlinear relationship between variables, which could be incorporated into the model after other additions [19,20]. In this study, penalized splines were applied to control the effects of confounding factors, such as long-term trends, seasonal trends and other long-term variables related to the time-series of daily deaths. The degree of freedom of the time trend was between 4 and 6. When the sum of the absolute value of partial correlation coefficients of the model residuals lagged by 1–2 days was less than 0.1, the basic condition of the model was satisfied. Otherwise, if the model was unsatisfied, self-correlated items were added to lower the sum of the absolute value, and the longest of the lag times was 7 days [21]. Dummy variables for day of week and festivals were also pulled into the model. The general form of the model was as follows:

(1) $$\log[E(Y_{t})]=\alpha +s(time,df.Time)+ns(Temp_{t},df.Temp)+ns(Humi_{t},df.Humi)+PM_{t}$$

In this model, *Y_t_* is the death number of day t; *E(Y_t_)* is the expected death number of day t; α is the intercept; s is the penalized splines; *df.Time*, *df.Temp* and *df.Humi* is the degree of freedom in the spline smoothing function of time trend, daily mean temperature and relative humidity, respectively; *PM_t_* is the mean concentration of PM_10_ of day t. The degrees of freedom of smooth splines were previously defined [22,23].

The exposure–response curve of the relationship between temperature and daily death was determined based on Model (1), and the temperature is further stratified for analysis by Model (2), as follows:

(2) $$\log[E(Y_{t})]=\alpha +s(time,df.Time)+Temp1_{t}+Temp2_{t}+Tempn_{t}+...+ns(Humi_{t},df.Humi)+PM_{t}$$

In this model, *Y_t_* is the death number of day t; *E(Y_t_)* is the expected death number of day t; α is the intercept; s is the penalized splines; *df.Time*, *df.Temp* and *df.Humi* is the degree of freedom in the Spline smoothing function of time trend, daily mean temperature and relative humidity, respectively; *PM_t_* is the mean concentration of PM_10_ of day t. Daily mean temperature *Tempn_t_* represents different temperature stratification levels.

We divided the temperature into low and high levels for stratified analysis to assess the acute effects of temperature. With reference to the threshold used in previous research and with most curves simulated in cities, we selected 21 °C as the temperature stratification level to study acute effects of different levels of temperature [18].

In this study, a distributed lag non-linear model (DLNM) was applied to estimate the lagged effect of temperature over 40 days on daily mortality according to previous literature [24]. For the multi-city data, meta-analysis was applied to merge the data. Taking into consideration the differences in climates among cities, random-effects models were used in the meta-analysis.

Results were presented as changes in excess risks (ER) with 95% confidence intervals (CI) in mortality for every 1 °C increase in temperature. The ER was calculated using Equation (3) as follows:

(3) ER=(RR−1)×100%

Where *RR* is relative risk of temperature on the mortality. All model analyses were performed using R software, version 3.0.3, using the mgcv package 1.6–2.

## 3. Results

### 3.1. Descriptive Analysis

The daily mean temperature of each city during 2007–2009 was 13.6 °C (Beijing), 13.3 °C (Tianjian), 18.0 °C (Xi’an), 6.1 °C (Harbin), 17.6 °C (Shanghai), 22.8 °C (Guangzhou) and 14.4 °C (Wuhan), respectively. Among these seven cities, the highest annual daily mean temperature was in Guangzhou. Harbin had the lowest annual daily mean ambient temperature (Table 2).

The daily non-accidental deaths of the residents in seven cities are also shown in Table 2, ranging from 18 to 175.

### 3.2. Acute Effects of Ambient Temperature on Daily Mortality

**Figure 1:**
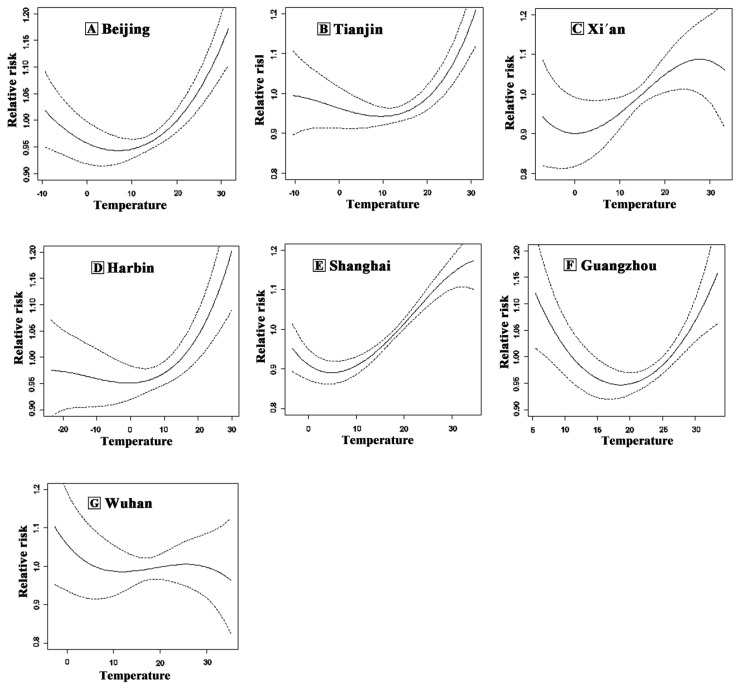
Exposure-response curve between temperature and non-accidental mortality for (**A**) Beijing; (**B**) Tianjin; (**C**) Xi’an; (**D**) Harbin; (**E**) Shanghai; (**F**) Guangzhou; and (**G**) Wuhan, China, 2007–2009. The solid lines represent the estimated relative risk in daily non-accidental mortality and the dotted lines represent the 95% confidence interval for each estimate.

At the single-city level, the exposure–response relationship in Beijing, Tianjin, Xi’an, Harbin, Shanghai and Guangzhou manifested a U-shape or V-shape, while the curve of Wuhan was an inverted J-shape (Figure 1). Considering this curvilinear relationship between ambient temperature and mortality, we might underestimate or overestimate the risk of death if it is simply included in the model as a linear factor. Thus, we divided the temperature into low and high levels for stratified analysis to assess its acute effects. With reference to the threshold used in previous research and with most curves simulated in cities, we chose 21 °C as the threshold to study acute effects, further facilitating the merger and analysis of data [18].

For non-accidental mortality, the excess risk of a high temperature in Beijing, Tianjin and Shanghai was significantly increased by 0.36% (95% CI: 0.11%, 0.62%), 0.43% (95% CI: 0.08%, 0.78%) and 0.75% (95% CI: 0.52%, 0.98%), respectively. The excess risk of a low temperature in Shanghai was significantly increased by 0.58% (95% CI: 0.33%, 0.82%). Overall, the excess risks of a high temperature for non-accidental mortality were all higher than those for a low temperature in Beijing, Tianjin, Xi’an, Harbin and Shanghai (Table 3).

### 3.3. Lagged Effects of Temperature on Non-Accidental Mortality

**Figure 2:**
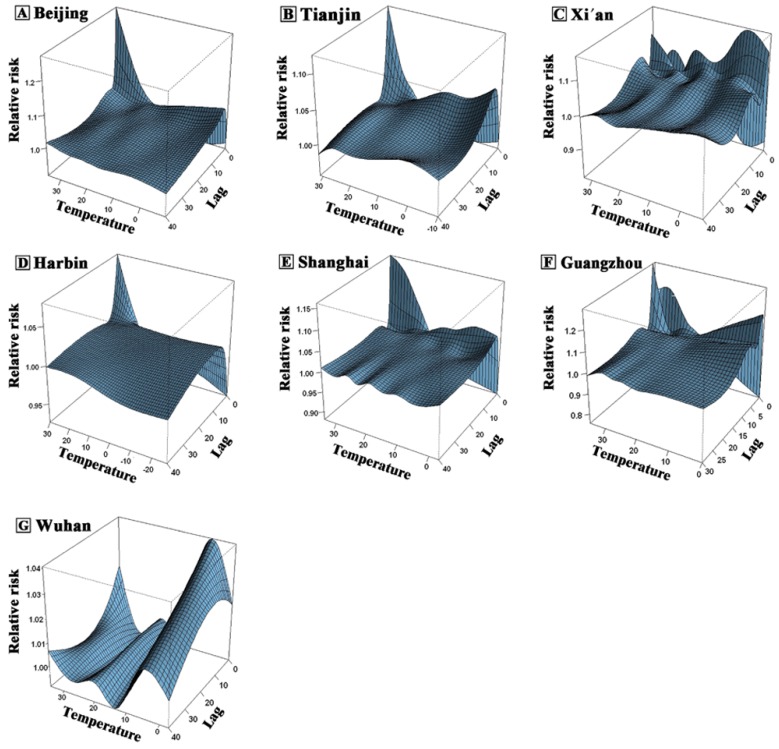
Three-dimensional plot of relative risk (RR) along temperature and lags on non-accidental mortality for (**A**) Beijing; (**B**) Tianjin; (**C**) Xi’an; (**D**) Harbin; (**E**) Shanghai; (**F**) Guangzhou; and (**G**) Wuhan, China, 2007–2009.

For most cities (Beijing, Tianjin, Harbin, Shanghai and Guangzhou), the lower the temperature, the longer the lag time. Usually there would be no significant effects within two weeks, and a strong effect appeared in 2–3 days. When the temperature was higher, the lag time was shorter. Generally, the duration was within one week with a strong effect in the first few days, and decreasing rapidly afterwards. After 38 days, none of the temperatures had a significant effect (Figure 2). For non-accidental mortality, no obvious lagged effects were observed for either high or low temperatures in Xi’an (Figure 2C). In Wuhan, when the temperature was low, the lag time was longer (even beyond one month), and a strong effect appeared in one week. When the temperature was high, no significant effects were observed (Figure 2G).

### 3.4. Optimum Temperature

**Figure 3:**
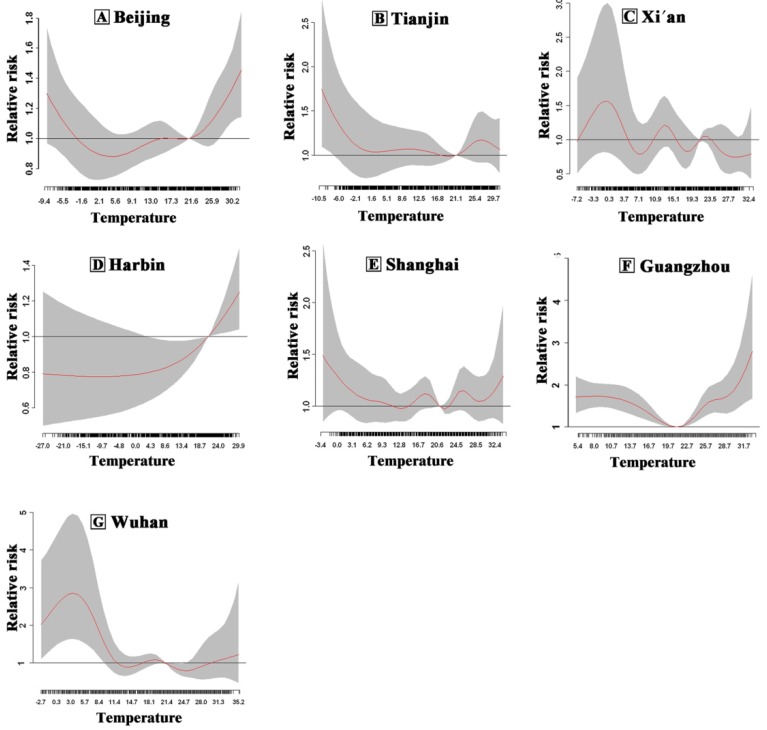
Overall plot of RR along temperature and lags on non-accidental mortality for (**A**) Beijing; (**B**) Tianjin; (**C**) Xi’an; (**D**) Harbin; (**E**) Shanghai; (**F**) Guangzhou and (**G**) Wuhan, China, 2007–2009. The red solid lines represent the estimated relative risk in daily non-accidental mortality and dash areas represent the 95% confidence interval for each estimate.

**Figure 4:**
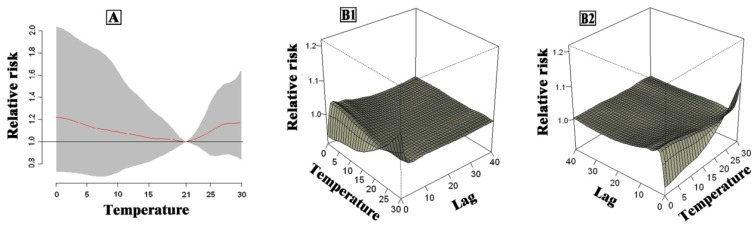
Overall (**A**) and three-dimensional (**B1** and **B2**) plot of RR in terms of temperature and lags and non-accidental mortality in combined cities.

The cumulative effect of a lagged effect of daily average temperature over 40 days in seven cities in China is shown in Figure 3. The cumulative effect for the exposure–response relationship of single city manifested “U“, “V“ or “W“ curves, and the optimum temperature was between 21 °C and 25 °C. The combined effect of the seven cities is shown in Figure 4. The relative risks of temperature on non-accidental mortality were presented as flat “U” curve with the lowest relative risk at 21 °C, which was the optimum temperature in the seven cities. 

## 4. Discussion

Due to global warming, the effect of temperature on health has aroused widespread concern. In recent years, more research has focused on the relationship between temperature and mortality. However, these studies are restrained by limitations of the research data. Because the research methods have been improved, the exposure–response relationship between temperature and mortality is an important issue in environmental epidemiology. There is a non-linear relationship in the “U-”, “V-” or “J-” curve reported between daily temperature and mortality. When the temperature was higher or lower than a critical temperature, mortality was expected to increase along with an increase or decrease of the temperature. However, this relationship is affected by multiple factors, such as geographical location, climate, socioeconomic status, and air pollution.

China is a developing country with the largest population in the world and a large land area. Thus, it is difficult to acquire a wide range of meteorological data and mortality-monitoring data, and data collection and statistical analyses must be precise. Thus, multi-city studies of the exposure–response relationship of temperature and mortality in China are rare. Between 2007 and 2009, the Chinese economy skyrocketed, and during this time, meteorological and mortality data collection were regulated and more reliable. The seven cities that we selected contain 55 million urban residents that reside in areas where atmosphere and mortality are well monitored. These cities also have the top monitoring systems in China ensuring data stability and reliability. In this study, we systematically investigated the exposure–response relationship of temperature and mortality using meteorology, air pollution and mortality-monitoring data from seven relatively developed cities in northern and southern China. 

In studies exploring the exposure–response relationship of temperature and mortality, choosing the temperature indicator is the first consideration, and there have been a few studies that discuss the merits of the effects of different temperature indicators on mortality. Metzger *et al.* [25] compared the heat index, and the maximum, minimum, and daily mean temperature using a goodness of fit equation in a study of the summer temperature–mortality relationship in New York. The result showed that the maximum heat index provided the best fit. However, Barnett *et al.* [26] used mortality data from 107 US cities for the years 1987–2000 to examine the association between temperature and mortality and found that there was no significant difference among the minimum, maximum, mean, and apparent temperature, and the heat index. Although previous studies have adopted the maximum and minimum temperature to study the relationship between temperature and mortality [27], daily mean temperature was the most commonly used indicator [10,28]. Taking into account that there is no clear evidence of which temperature indicator is best, we chose the daily mean temperature as the indicator in our study, which is the most broadly understood temperature indicator.

The shape of the temperature–mortality curve in different cities may be very different. Taking into consideration the significant interaction between outdoor air pollution and temperature [29], in our single city experiment, we adjusted PM_10_ as a confounding factor. Also, the relationship between temperature and mortality in most cities exhibited a “U-” or “V-” curve, while few cities showed an “inverted-J” curve. This result may have been affected by many factors, including geographical position, air pollution levels, living habits and socio-economic status, *et al.* Thus, the merged result of multi-cities could be relatively stable [10,30]. After combining the results from multi-city analysis, the exposure–response relationship showed flat "U" curve, which is quite consistent with previous multi-city studies in China [18,31].

In the study of the exposure–response relationship between temperature and mortality, the temperature is generally divided as high and low to study its effect. Our study demonstrated that high temperature posed a higher risk of an acute effect on mortality than low temperature, which is consistent with the multi-city studies from America, Europe and China [18,32,33,34]. Medina-Ramon explored the effects of extreme temperatures on non-accidental deaths in 1989–2000 in 42 U.S. cities via case-crossover, which defined a temperature ≤ 1st percentile and ≥ 99th percentile as extreme cold and extreme hot. This study found that the risk of extreme heat and extreme cold temperatures on mortality was 5.74% (95% CI: 3.38, 8.15) and 1.59% (95% CI: 0.56, 2.63), and high temperature posed a greater risk of mortality [32]. Iniguez *et al.* studied the relationship between temperature and death in 13 cities in Spain during 1990–1996. They used MMT (minimum mortality temperature) to divide high and low temperature, and showed that the risk of high temperature on mortality was higher than the low temperature [33]. Ma *et al.* [18] studied the relationship between temperature and death in 17 Chinese cities, and reported that the risk of mortality for hot temperatures was higher than the risk for cold temperatures. The effects of high and low temperature in this study were both stronger than our results, probably due to the difference in the cities selected and the study periods, as well as uncontrolled confounding factors (e.g., air pollution, which was controlled in our study).

The lagged effect of temperature on mortality has received a lot of attention, which could further reveal the features of the exposure–response relationship between temperature and mortality. In our study, DLNM was applied to study the lagged effect of temperature over 40 days. It should be noted that there may be strong co-linearity between the different temperatures of the lagged days in the polynomial distributed lag model, which could lead to unstable lag estimates. In our study, the degree of freedom of coefficient in the model was restricted to be more flexible, and bias was minimized [10,35]. Consistent with previous studies, our results found the effect of high temperature was short, lasting generally less than one week, while the effect of low temperature was long, lasting almost two weeks. Baccini *et al.* studied the relationship between maximum apparent temperature and mortality in 15 European cities during April to September from 1990 to 2000 using five rank distribution lag models to study a lagged effect of 40 days. The results showed that the lagged effect of the maximum apparent temperature manifested within the first week [36]. Yu *et al.* [37] studied the lagged effect of temperature on cardiovascular disease mortality in Brisbane, Australia during 1996–2004. A lagged effect of 30 days was investigated using a polynomial distributed lag model, which suggested that the effects of low temperature lasted longer. Ma *et al.* [18] estimated the relationship between temperature and non-accidental mortality in 66 Chinese communities using the DLNM. The results showed that high temperature had a higher effect in North China than in South China. The different duration of the effects of low and high temperature may indicate that low and high temperatures initiate different mechanisms. Regarding high temperature, a decreased risk may be caused by adaption to heat exposure and/or a decrease of the susceptible population [38]. When the temperature is low, the number of platelets, red blood cells, blood viscosity, blood pressure and the production of reactive oxygen species may be increased, which would cause oxidative damage [39]. As exposure time increases, the human body increases the ability to remove free radicals and gradually restores the normal physiological state [40,41,42,43].

Exploring the optimum environmental temperature for a population is relevant for safe and healthy travel and for the regulation of indoor temperature. A large number of epidemiological studies have confirmed that there is a correlation between temperature and short-term mortality. It is feasible to explore the optimum temperature for the population through the study of this relationship. Our multi-study results showed that the exposure–response relationship of temperature and non-accidental mortality manifested “U” curve. In the cut-off point of the “U” curve, the risk posed by temperature on mortality was at a minimum, which is the optimum temperature. The optimum temperature could be a temperature point or a range of temperatures, while our study identified 21 °C as the optimum temperature. The optimum temperature could vary in different geographical locations and climates. Several studies have reported different optimum temperatures in different regions or countries. The optimum temperature in London, United Kingdom was 19 °C [44], in the Netherlands, 16.5 °C [45], while in Valencia, Spain the optimum temperature was 22–25 °C [46]. A multi-city study in China showed that the optimum temperature was 17.7 °C in the Northeast, 19.5 °C in the North, 18.2 °C in the Northwest, 23.4 °C in the East, 21.1 °C in the Central part, 26.5 °C in the Southwest and 27.4 °C in South China [18]. This heterogeneity may be owing to the difference of the latitude of the cities and the lifestyle of residents in the cities [47]. Furthermore, these differences may influence the physiological adaptation and behavioral adaptation of the population. People may be able to adapt to long-term climate conditions within a certain limit. When the ambient temperature is above or below a certain temperature range or threshold, the body’s heat balance adjustment mechanism may be disrupted if the body is too hot or too cold. The burden of physiological thermoregulation would be increased, thus causing discomfort, exacerbation and even death. So, in facing climate change, our study suggests that the public should keep indoor temperature within an optimum temperature range as much as possible to prevent the potential adverse effects of extreme temperature.

Our study has several limitations. First, our study mainly focuses on seven major Chinese cities with strong economies, while other cities in China with relatively low economies were not included. Thus, our results cannot be generalized to any city in China. Second, the mortality outcomes did not account for age, sex, disease, social conditions and other factors. These factors could modify the exposure–response relationship between temperature and mortality.

Our study also has several strengths. Firstly, this study is based on the data of multi-Chinese typical large cities with a population of more than 55,000,000, which could provide evidence for the spatial and population distribution of temperature–mortality relationship of China. Secondly, the lagged effects of temperature up to 40-days on mortality were analyzed which could guarantee the accuracy of the results of optimum temperature. Finally, sophisticated statistical models were applied to estimate the temperature–mortality relationship of multi-cities in China, and several confounding factors such as air pollution, relative humidity, long-term trends and seasonal trends were adjusted in the models.

## 5. Conclusions

Multi-city studies focusing on the exposure–response features of temperature and mortality in China are rare. In this study, we used GAM and DLNM to explore the exposure–response features of temperature and mortality based on monitoring data from seven large Chinese cities. There was an acute effect of ambient temperature change on population mortality, and high temperature posed a greater risk of mortality compared to low temperature. We observed a lagged effect between ambient temperature change and mortality in a multi-city analysis of cities in China of about one week for high temperature and two weeks for low temperature. The optimum temperature for the Chinese population was 21 °C. These findings could be useful for providing guidance for safe travel, the reasonable adjustment of ambient room temperature, and the design of public health policy.

## Figures and Tables

**Table 1:** Summary description of the population and the districts of seven large cities in China.

City	Number of Districts	Name of Districts	Population (× 10^4^)
Beijing	14	Dongcheng, Xicheng, Chaoyang, Fengtai, Shijingshan, Haidian, Mentougou, Fangshan, Tongzhou, Shunyi, Changping, Daxing, Huairou, Pinggu	1620.60
Tianjin	11	Hedong, Hexi, Heping, Nankai, Hebei, Hongqiao, Dagang, Tanggu, Dongli, Beichen, Hangu	724.11
Xi'an	6	Xincheng, Beilin, Lianhu, Baqiao, Weiyang, Yanta	450.57
Harbin	6	Daoli, Nangang, Daowai, Pingfang, Songbei, Xiangfang	354.67
Shanghai	10	Huangpu, Luwan, Xuhui, Changning, Jingan, Putuo, Zhabei, Hongkou, Yangpu, Pudongxin	958.07
Guangzhou	8	Liwan, Yuexiu, Haizhu, Tianhe , Baiyun, Huangpu, Fanyu, Huadu	845.01
Wuhan	8	Jiangan, Jianghan, Qiaokou, Hanyang, Wuchang, Qingshan, Hongshan, Dongxihu	600.70
Total	63		5553.73

Data source: Statistics yearbook 2009 of each city in China.

**Table 2:** Summary statistics of the variables of seven large cities in China.

City	Variables	Min.	P(25)	Median	P(75)	Max.	Mean ± SD
Beijing	temperature(°C)	−9.4	3.0	15.1	24.1	31.6	13.6 ± 10.9
	humidity (%)	11	36	53	69	97	52.5 ± 20.2
	PM_10_	7	72	116	160	600	130.7 ± 81.9
	mortality	107	152	171	193	260	175 ± 28
Tianjin	temperature(°C)	−10.5	2.6	14.6	23.9	31	13.3 ± 11.2
	humidity (%)	15	45	60	73	95	58.3 ± 18.3
	PM_10_	13	58	80	118	503	93.9 ± 55.4
	mortality	56	90	100	113	175	101 ± 17
Xi'an	temperature(°C)	−2.7	9.8	19.4	26.1	35.3	18.0 ± 9.4
	humidity (%)	21	61	70	79	97	69.6 ± 13.0
	PM_10_	18	72	106	144	567	112.9 ± 54.4
	mortality	7	20	27	34	63	28 ± 10
Harbin	temperature(°C)	−27	−7.4	8.6	19.7	29.9	6.1 ± 14.4
	humidity (%)	17	51	63	72	96	61.1 ± 15.7
	PM_10_	12.6	62	84	124	600	99.6 ± 62.2
	mortality	40	76	87	99	138	88 ± 16
Shanghai	temperature(°C)	−3.4	9.8	18.7	25.2	34.6	17.6 ± 8.9
	humidity (%)	30	62	71	79	95	69.7 ± 12.1
	PM_10_	12	48	73	106	600	84.0 ± 52.5
	mortality	70	96	106	119	167	108 ± 17
Guangzhou	temperature(°C)	5.4	18.3	24.6	27.8	33.5	22.8 ± 6.3
	humidity (%)	25	63	71	81	94	70.6 ± 13.3
	PM_10_	7	43	63	92	297	72.8 ± 41.4
	mortality	17	37	51	61	106	51 ± 16
Wuhan	temperature(°C)	−7.2	5.8	15.5	23	33.3	14.4 ± 9.7
	humidity (%)	19	52	66	78	100	64.9 ± 17
	PM_10_	29	82	114	144	556	121.3 ± 55.2
	mortality	1	12	17	22	49	18 ± 7

PM_10_ = particulate matter with an aerodynamic diameter of less than 10 μm, SD = standard deviation.

**Table 3:** Acute effects of ambient temperature on non-accidental mortality in different levels of seven large cities in China.

City	Temperature	Excess Risk (%)
Beijing	high (>21 °C)	0.36 (0.11, 0.62) *
	low (<21 °C)	0.25 (0.00, 0.51)
Tianjin	high (>21 °C)	0.43 (0.08, 0.78) *
	low (<21 °C)	0.28 (−0.09, 0.65)
Xi'an	high (>21 °C)	0.45 (−0.10, 1.00)
	low (<21 °C)	0.43 (−0.15, 1.03)
Harbin	high (>21 °C)	0.23 (−0.05, 0.51)
	low (<21 °C)	0.14 (−0.13, 0.42)
Shanghai	high (>21 °C)	0.75 (0.52, 0.98) *
	low (<21 °C)	0.58 (0.33, 0.82) *
Guangzhou	high (>21 °C)	−0.03 (−0.45, 0.38)
	low (<21 °C)	−0.17 (−0.67, 0.32)
Wuhan	high (>21 °C)	−0.16 (−0.76, 0.45)
	low (<21 °C)	−0.5 (−1.16, 0.15)
Overall	high (>21 °C)	0.33 (0.11, 0.56) *
	low (<21 °C)	0.21 (−0.05, 0.48)

Notes: Data presented as % (95% *CI*). * *p* < 0.05 (the association was significant). Overall: seven cities (Beijing, Tianjin, Xi'an, Harbin, Shanghai, Guangzhou and Wuhan).
